# Supplementary material for: COVID-19 preventive practices during intrapartum care- adherence and barriers in Ethiopia; a multicenter cross- sectional study
Source: PLoS One. 2021 Nov 18;16(11):e0260270. doi: 10.1371/journal.pone.0260270 (PMC8601578; doi:10.1371/journal.pone.0260270)
Supplement: S1 Questionnaire — (DOCX) [file pone.0260270.s001.docx]

Annex1: English version of the questionnaire

Part I: Socio-demographic characteristics

| N.O | Questionnaire | Alternative choice for response |
| --- | --- | --- |
| 101 | How old are you? | (In years) |
| 102 | What is your sex? | 1. Male 2. 2. Female |
| 103 | Professional category | 1. Midwifery-Diploma 2. Midwifery-Degree 3. Midwifery-Master’s degree 4. Medical interns 5. IESO 6. Other specify |
| 104 | What is your marital status? | 1. Married  2. Unmarried |
| 105 | What is your average monthly income? | (In ETB) |
| 106 | Have you ever read newspapers? | 1. Yes 2. 2. No |
| 107 | Have you ever watched a television? | 1. Yes 2. 2. No |
| 108 | If yes, how often did you watch? | 1. Every day 2. One times per week 3. Two times per week 4. Three and more |
| 109 | Do you have a smartphone or computer | 1. Yes 2. No |

Part II: **Workplace profession related questions**

| NO | Questioners | Alternative choices for response |
| --- | --- | --- |
| 201 | Experiences in years (months) |  |
| 202 | Self-rated relation with the nearby boss? | 1. Good  2. Poor |
| 203 | Job satisfaction related variables |  |
|  | Are you satisfied in your payment | 1. Yes 2. No |
|  | Are you satisfied related to promotion | 1. Yes 2. No |
|  | Are satisfied by the supervision process of your organization | 1. Yes 2. No |
|  | Are you satisfied by your coworkers | 1. Yes 2. No |
|  | Are you satisfied by rewards and benefits in your organization | 1. Yes 2. No |
|  | Are you satisfied by the nature of your work | 1. Yes 2. No |
| 204 | Facility type | 1. Primary hospital  2. General hospital  3. Tertiary hospital |
| 205 | When was the working time (the time during data collection)? | 1. 1. Night 2. 2. Day |
| 206 | Facility location | 1. Urban  2. Semi-urban |
| 207 | Are you intended to stay in the profession for the future? | 1. Yes  2. No |
| 208 | Is there workload in the hospital and/or shortage of staffs? | 1. 1. Yes 2. 2. No |
| 209 | Are you interested to work in the delivery unit? | 1. 1. Yes 2. 2. No |
| 210 | Do you work part-time at a private health facility? | 1. Yes 2. 2. No |
| 211 | Do you have an education to learn while working (like, BSc, MSc by your own)? | 1. Yes 2. No |
| 212 | Have ever received training on infection prevention? | 1. Yes 2. No |

**Part VII- Providers adherence to COVID-19 prevention protocols during intrapartum care (observation)**

| No | Questions | Answer |
| --- | --- | --- |
| 701 | Did the health care provider wash his/her hand before examining the laboring women? | 1. Yes  2. No |
| 702 | Did the health care provider use alcohol based hand rub before touching the laboring women? | 1. Yes  2. No |
| 703 | Did the health care provider wear facemask while caring the women? | 1. Yes  2. No |
| 704 | Did the health care provider wear protective eyewear/ splash guard while caring the women? | 1. Yes  2. No |
| 705 | Did the health care provider wear protective gowns or work uniform while caring the women? | 1. Yes  2. No |
| 706 | Did the health care provider wear disposable glove during examining each woman? | 1. Yes  2. No |
| 707 | Did the health care provider wear disposable surgical cap? | 1. Yes  2. No |
| 708 | Did the health care provider limit number of visitors during labor and delivery? | 1. Yes  2. No |
| 709 | Did the health care provider properly remove used/contaminated materials in the appropriate place? | 1. Yes  2. No |
| 710 | Did the health care provider wash his/her hand after touching the women? | 1. Yes  2. No |
| 711 | Did the health care provider shakes hands of any individual in the maternity ward? | 1. Yes  2. No |
| 712 | Did the healthcare provider avoid touching his eye, nose and mouth with unwashed hand? | 1. Yes  2. No |
| 713 | Did the healthcare provider keep contact with others? | 1. Yes  2. No |
| **Barriers for not follow the COVID-19 prevention protocols during childbirth** | | |
| **Why health care providers didn’t follow the COVID-19 prevention protocols?**  Overcrowding of the delivery room (case overload)  Less commitment or negligence of healthcare providers  Lack of clear information regarding COVID-19 prevention protocol  Lack of clear policy and procedures in the delivery unit regarding COVID-19 prevention  Lack of water (non-functional ) in the delivery unit  Lack of face mask and other personal protective equipment’s  Non availability of alcohol or sanitizer  Believing COVID-19 preventing methods may not be effective  Other specify | | |
