# Supplementary material for: COVID-19 preventive practices during intrapartum care- adherence and barriers in Ethiopia; a multicenter cross- sectional study
Source: PLoS One. 2021 Nov 18;16(11):e0260270. doi: 10.1371/journal.pone.0260270 (PMC8601578; doi:10.1371/journal.pone.0260270)
Supplement: S1 File — (DOCX) [file pone.0260270.s003.docx]

List of the full names of the selected health hospitals for this study

1. University of Gondar comprehensive specialized hospital
2. Debre Tabor specialized hospital
3. Debark General hospital
4. Ambagiorgis primary hospital
5. Dembia primary hospital
6. Metema primary hospital
7. Tach Giant primary hospital
8. Lay Gaint primary hospital
9. Gohala primary hospital
10. Ebinat primary hospital
11. Andabet primary hospital
12. Delgi primary hospital
13. Ayikel primary hospital
14. Mekaneyesus primary hospital
15. Addis Zemen primary hospital
