# Supplementary material for: COVID-19 preventive practices during intrapartum care- adherence and barriers in Ethiopia; a multicenter cross- sectional study
Source: PLoS One. 2021 Nov 18;16(11):e0260270. doi: 10.1371/journal.pone.0260270 (PMC8601578; doi:10.1371/journal.pone.0260270)
Supplement: Attachment — Submitted filename: Response to Reviewers.docx [file pone.0260270.s004.docx]

**July 4, 2021**

**Manuscript ID:** PONE-D-21-09407

**Response to Reviewers**

First of all, we would like to acknowledge your constructive comments on and valuable improvements to our paper. We have incorporated the suggestions and comments with tracked changes in the revised manuscript and a point-by-point response, please see below.

**Response to Editor Comments**

| S.NN | Comments | Responses |
| --- | --- | --- |
| 01 | Please ensure that your manuscript meets PLOS ONE's style requirements, including those for file naming | Thank you very much, we have revised the manuscript as per your kind recommendation; PLOS ONE style template. |
| 02 | Please include your actual numerical p-values in Table 4 | Thank you dear editor, we have included the actual p-values in the revised manuscript. |
| 03 | Please provide a list of the full names of the selected health hospitals for this study (you may provide this as a supplementary file). | Thank you dear editor, we have included the selected hospitals in the method section (sampling technique section with the respective sample size for each hospital) |
| 04 | Please include captions for your Supporting Information files at the end of your manuscript, and update any in-text citations to match accordingly | Thank you very much, we did that in the revised manuscript and we incorporate all the required supporting information |
| 05 | The academic editor served as the second reviewer on this manuscript and agree that major revisions, especially methods, needs to be addressed for further publication consideration. | We are very glad to thank the academic editor for his time, insight full comments, and suggestions. We tried to make the method section statically rigorous in the revised manuscript. Thank you once again for your time dear editor. |

**Response to Reviewer(s) comments**

**Reviewer #1**

| S.N | Comments | Responses |
| --- | --- | --- |
| 01 | Objective- It is important to assess whether COVID-19 pandemic prompted improvement or change in infection practice during labour and childbirth. This can be done through observation before and during COVID-19 pandemic. I see that the study collected data through observation on infection prevention practice during pandemic. This is one of the limitation of the study. Can the researcher collect data on maternal and neonatal infection rate in these hospitals before and during pandemic? | Thank you dear reviewer for your insightful comments and deep review. We really appreciate the concern and we were thinking of the issue. However, we couldn’t do that due to feasibility issues. So, the main aim of the study was whether healthcare providers are following the COVID-19 prevention practices during childbirth that are recommended by the World Health Organization (WHO). Because the maternity ward is overcrowded and stressful by its nature, and health professionals may not strictly follow the WHO COVID-19 preventive practice recommendations because they might be worried about the health of the mother and the unborn baby. We didn’t plan to assess the maternal and neonatal infection rate rather, we intended to assess whether healthcare providers properly follow the COVID-19 preventive methods while they are working in the labor ward. |
| 02 | Design- Can researcher make it a retrospective cohort study, with change in infection rate before and during pandemic. | Thank you for your kind recommendation, but as we described above the aim of this study was to assess the healthcare provider’s COVID-19 prevention practice at a point during intrapartum care. It is a cross-sectional observational study. We make it an observational study so as to minimize the response and social desirability bias that is commonly encountered in web-based and self-administered studies. |
| 03 | Data collection- One key variable of interest is did the infection prevention practice change by obstetric characteristics (mode of birth, complication during labour). Can the data collection be made on mode of birth? Further, the data collection on the continuous supply of PPE is important, can research provide the supply chain system. | Thank you dear reviewer for your interesting viewpoint. However, we missed collecting the aforesaid data as our study primarily focused on variables related to the healthcare providers. Because, if we would understand the recommendation, the mode of delivery or childbirth-related complications were not our primary concerns. Regardless of the aforementioned factors, we intended to assess the healthcare provider’s adherence to COVID-19 preventive practice while doing their work in the maternity ward. That means our concern was the healthcare provider’s adherence level to the routine COVID-19 preventive practices regardless of other maternal and obstetrical characteristics. |
| 04 | Data analysis- Can the inter-hospital infection prevention practice variance be provided | Thank you dear reviewer, it was possible to provide the inter-hospital infection prevention variance, however, we didn’t assess the inter-hospital infection prevention practice variance to make the data anonymous and keep the confidentiality of the institutions as well as health professionals. Dear reviewer, however, we really appreciate your kind recommendation. |
| 05 | So, what the policy implication of the study | Thank you for your kind recommendation, we included the implications of the study for policymakers at the discussion part in the revised manuscript. |
| 06 | There are typos in the research, if accepted needs a thorough English language review | Thank you dear reviewer, we tried to look for the English language in the revised manuscript. |
