# Supplementary material for: COVID-19 preventive practices during intrapartum care- adherence and barriers in Ethiopia; a multicenter cross- sectional study
Source: PLoS One. 2021 Nov 18;16(11):e0260270. doi: 10.1371/journal.pone.0260270 (PMC8601578; doi:10.1371/journal.pone.0260270)
Supplement: Attachment — Submitted filename: Response to reviewers..docx [file pone.0260270.s005.docx]

**September 1, 2021**

**Manuscript ID:** PONE-D-21-09407R1

**Response to Reviewers**

First of all, we would like to acknowledge your constructive comments on and valuable improvements to our paper. We have incorporated the suggestions and comments with tracked changes in the revised manuscript and a point-by-point response, please see below.

**Response to Editor (journal requirements) Comments**

| S.NN | Comments | Responses |
| --- | --- | --- |
| 01 | Please review your reference list to ensure that it is complete and correct.  If you have cited papers that have been retracted, please include the rationale for doing so in the manuscript text, or remove these references and replace them with relevant current references. | Thank you, we thoroughly review the references and there is retracted paper cited. However, we added one reference (reference 18) based on the recommendation from the reviewer. |
| 02 | The academic editor served as the second reviewer on this manuscript and agree that major revisions, especially methods, needs to be addressed for further publication consideration. | We are very glad to thank the academic editor for his time, insight full comments, and suggestions. We tried to make the method section statically rigorous in the revised manuscript. Thank you once again for your time dear editor. |

**Response to Reviewer(s) comments**

**Reviewer #1**

| S.N | Comments | Responses |
| --- | --- | --- |
| 01 | The title needs to be shortened can it be "COVID-19 preventive practices during intrapartum care-adherence and barriers in Ethiopia; a multi-centered cross-sectional study | Thank you, dear reviewer, we did a modification on the title based on your kind recommendation. You can see it in the revised manuscript. |
| 02 | In the abstract, findings please remove word "statistically" as significant association does imply that. | Thank you, we removed the word “statistically” from the abstract section. |
| 03 | Please add the reference on "Perfect storm by KC A et al" <http://www.jogh.org/documents/2021/jogh-11-05010.htm> which shows improvement in infection prevention practices. | Thank you, we added this reference in the introduction section (reference 18). Overall, we are thankful for your scientific contribution and important addition to our paper. |
