# Supplementary material for: COVID-19 preventive practices during intrapartum care- adherence and barriers in Ethiopia; a multicenter cross- sectional study
Source: PLoS One. 2021 Nov 18;16(11):e0260270. doi: 10.1371/journal.pone.0260270 (PMC8601578; doi:10.1371/journal.pone.0260270)
Supplement: Attachment — Submitted filename: Response to reviewers....docx [file pone.0260270.s006.docx]

**October 7, 2021**

**Manuscript ID:** PONE-D-21-09407R2

**Response to Reviewers**

First of all, we would like to acknowledge your constructive comments on and valuable improvements to our paper. We have incorporated the suggestions and comments with tracked changes in the revised manuscript and a point-by-point response, please see below.

**Response to Editor (journal requirements) Comments**

| S.NN | Comments | Responses |
| --- | --- | --- |
| 01 | Please review your reference list to ensure that it is complete and correct.  If you have cited papers that have been retracted, please include the rationale for doing so in the manuscript text, or remove these references and replace them with relevant current references. | Thank you, we thoroughly reviewed the references and there is no retracted paper cited. |
| 02 | The academic editor served as the second reviewer on this manuscript and agree that minor revisions. | We are very glad to thank the academic editor for their time, thought full comments, and suggestions for the ultimate improvement of the manuscript. |

**Response to Reviewer (s) comments**

**Reviewer #1**

| S.N | Comments | Responses |
| --- | --- | --- |
| 01 | As I revisited the manuscript and result, I noted that health care provider who earn better has been found to be associated with better care. This is a confounder, since health worker who earn more might be better professionally educated such as specialist doctor. So, I suggest to assess the association between education and control earning variable. I suggest to conduct the association between training in table 2 and conduct either multi variate or multi-level association with care i.e. in table 4. | We would like to thank the reviewer for their effort, continuous commitment, and timely response to the comments. We found all the comments raised during the review process are important. Yes, you are right, dear reviewer, higher monthly income and higher specialty might have a compounding effect. However, we did a multicollinearity test and it was acceptable. Besides, there were no senior specialists as indicated in table 1; general practitioners and residents were included. This is due to, most of the time senior specialists are consultants who might not be present in the maternity ward frequently unless consulted. So, There might not have visible income difference between senior midwives, residents, and general practitioners. Moreover, a multicollinearity test has been performed and a sentence describing it has been included in the **data processing and analysis** section---please see in the revised manuscript. |
| 02 | The reference in 53 to explain why health workers earning more with better intrapartum care, just not well explain the association | Thank you, dear reviewer, it was not to mean earning more was associated with better intrapartum care, rather this reference was cited to support the finding in which health professionals having better income was associated with general COVID-19 prevention practices. That means earning more income was enabling factors for healthcare providers in the general healthcare system and during CROVD-19 as well |
| 03 | Further, the methodological consideration is not provided, please kindly provide the limitation of the study especially the design. | Dear reviewer, you gave us very vital suggestion which is important for a research. We gratefully accepted and included it in the updated paper just after the discussion section-please see the revised manuscript. |
